# PEDV envelope protein promotes viral replication by remodeling host iron homeostasis via the TRIM28-KLF15-FPN axis

**DOI:** 10.1128/jvi.01239-26

**Published:** 2026-09-02

**Authors:** Leyu Hu, Ru Tian, Lexi Gao, Ning Xu, Peizhen Li, Lijie Wang, Xingwei Fu, Zesheng Rong, Muzi Du, Yixuan Ma, Yue Cheng, Lu Han, Xin Ran, Guiwen Guan, Ting Zhou, Lingling Chang, Xiaomin Zhao, Qian Du, Yong Huang, Dewen Tong, Mingjun Zhu

**Affiliations:** 1 College of Veterinary Medicine, Northwest A&F University 12469, Yangling, China; 2 Engineering Research Center of Efficient New Vaccines for Animals, Ministry of Education, Yangling, China; 3 Key Laboratory of Ruminant Disease Prevention and Control (West), Ministry of Agriculture and Rural Affairs, Yangling, China; 4 Engineering Research Center of Efficient New Vaccines for Animals, Universities of Shaanxi Province, Yangling, China; Loyola University Chicago - Health Sciences Campus, Maywood, Illinois, USA

**Keywords:** PEDV, FPN, KLF15, TRIM28, ubiquitination, iron accumulation

## Abstract

Porcine epidemic diarrhea virus (PEDV), a member of the Alphacoronavirus genus, causes severe enteric disease with up to 100% mortality in suckling piglets. Iron is an essential micronutrient for the replication of various pathogens, including viruses, which hijack host iron resources to support their proliferation. *SLC40A1* encodes ferroportin (FPN), the only known iron exporter in mammals and a key target for pathogens to manipulate host iron homeostasis. However, whether coronaviruses exploit this mechanism remains unknown. Here, we demonstrate that PEDV infection disrupts host iron homeostasis through the KLF15-FPN regulatory axis, resulting in suppressed FPN expression, intracellular iron accumulation, and enhanced viral replication. Mechanistically, the PEDV envelope (E) protein induces cytoplasmic aggregation of Kruppel-like factor 15 (KLF15) through its PDZ-binding motif (PBM), promoting tripartite motif-containing protein 28 (TRIM28)-dependent K63-linked ubiquitination and autophagy-lysosomal degradation of KLF15 at lysine 339 (K339), thereby suppressing KLF15-mediated transcriptional activation of *SLC40A1*. Our findings uncover a novel iron hijacking strategy by which PEDV remodels host iron homeostasis through the KLF15-FPN regulatory axis, providing new insights into coronavirus-host interactions and potential antiviral intervention strategies.

**IMPORTANCE:**

Porcine epidemic diarrhea virus (PEDV) causes a highly contagious enteric disease, posing a persistent threat to global swine production. Iron serves as a critical cofactor in numerous host and viral enzymatic processes, and profoundly influences viral infection outcomes. Ferroportin (FPN) is currently the only known iron excretory protein in mammals and has emerged as a key regulator of host iron homeostasis that can be exploited by pathogens. However, whether and how coronaviruses employ host iron regulatory pathways to remodel iron homeostasis remains unclear. Here, we reveal that PEDV E protein induces intracellular iron accumulation via the TRIM28-KLF15-FPN signaling axis, uncovering a novel function of this viral protein. This study advances our understanding of PEDV pathogenesis and highlights potential opportunities for developing anti-coronavirus strategies targeting host iron regulation.

## INTRODUCTION

Coronaviruses (CoVs) are major zoonotic pathogens that affect both humans and animals and comprise four genera: Alphacoronavirus, Betacoronavirus, Gammacoronavirus, and Deltacoronavirus. Representative members include porcine epidemic diarrhea virus (PEDV, alpha), severe acute respiratory syndrome coronavirus 2 (SARS-CoV-2, beta), infectious bronchitis virus (gamma), and porcine deltacoronavirus (delta) (1). Of these, PEDV contains a single-stranded, positive-sense RNA genome of approximately 28 kb, encoding 16 nonstructural proteins (nsp1–nsp16), 4 structural proteins, namely the spike (S) protein, envelope (E) protein, membrane (M) protein, and nucleocapsid (N) protein, and 1 accessory protein, ORF3 (2). PEDV causes porcine epidemic diarrhea, a highly contagious enteric disease characterized by vomiting, watery diarrhea, and dehydration, with mortality reaching up to 100% in suckling piglets (3, 4). Since its first report in the UK in the 1970s, PEDV has spread globally. Highly virulent PEDV variants emerged in China in 2010 and rapidly disseminated worldwide (5, 6). However, existing vaccines provide limited protection against currently circulating PEDV variants, underscoring the need to better understand the molecular mechanisms underlying PEDV pathogenesis (7).

Iron is an essential trace element that plays diverse roles in cellular metabolism. It serves as a critical cofactor for numerous enzymes and is indispensable for nucleic acid synthesis and mitochondrial energy production (8). As a key constituent of mitochondrial respiratory chain complexes, cytochromes, and iron-sulfur cluster proteins, iron is central to maintaining cellular energy homeostasis (9, 10). Beyond these fundamental roles, iron is also critically involved in viral replication. Many viruses exploit host cellular iron metabolism to create a cellular environment favorable for their life cycles, in part because iron supports the activity of host or virus-induced enzymes involved in nucleotide synthesis and energy metabolism (11). Iron availability can therefore influence viral replication efficiency, with iron deficiency restricting replication of certain viruses and iron overload favoring viral propagation and exacerbating pathogenesis (12–14). Thus, iron homeostasis represents a pivotal determinant of viral infection outcome.

Cellular iron homeostasis is maintained through coordinated iron uptake, storage, utilization, transport, and efflux. Iron uptake is mediated by transferrin receptor (TFR) and divalent metal transporter 1 (DMT1), whereas ferroportin (FPN), encoded by *SLC40A1*, serves as the only known iron exporter in mammals (15). FPN regulates the intracellular labile iron pool, supporting iron-dependent metabolism while preventing iron overload-induced oxidative damage (16). Dysregulated FPN expression is closely linked to iron-related disorders, such as hereditary hemochromatosis, iron-loading anemia, and anemia of chronic disease, and has emerged as a key target exploited by pathogens to alter intracellular iron availability (17–19). Notably, herpes simplex virus type 1 (HSV-1), vesicular stomatitis virus (VSV), and Sendai virus (SeV) downregulate FPN via DTX3L-mediated K48-linked ubiquitination and proteasomal degradation, leading to intracellular iron accumulation and enhanced viral replication (20). However, whether and how coronaviruses, particularly PEDV, manipulate the FPN-centered iron-export pathway to acquire host iron remains unknown.

In this study, we uncover a previously unrecognized mechanism by which PEDV disrupts host iron homeostasis to facilitate viral replication. Specifically, we demonstrate that the PEDV E protein induces cellular iron accumulation through the TRIM28-KLF15-FPN axis. Our findings uncover a novel nutritional hijacking strategy by which PEDV enhances its replication through inhibition of cellular iron export, offering new insights into coronavirus-host interactions.

## RESULTS

### PEDV downregulates FPN expression both *in vivo* and *in vitro*

**Fig 1:**
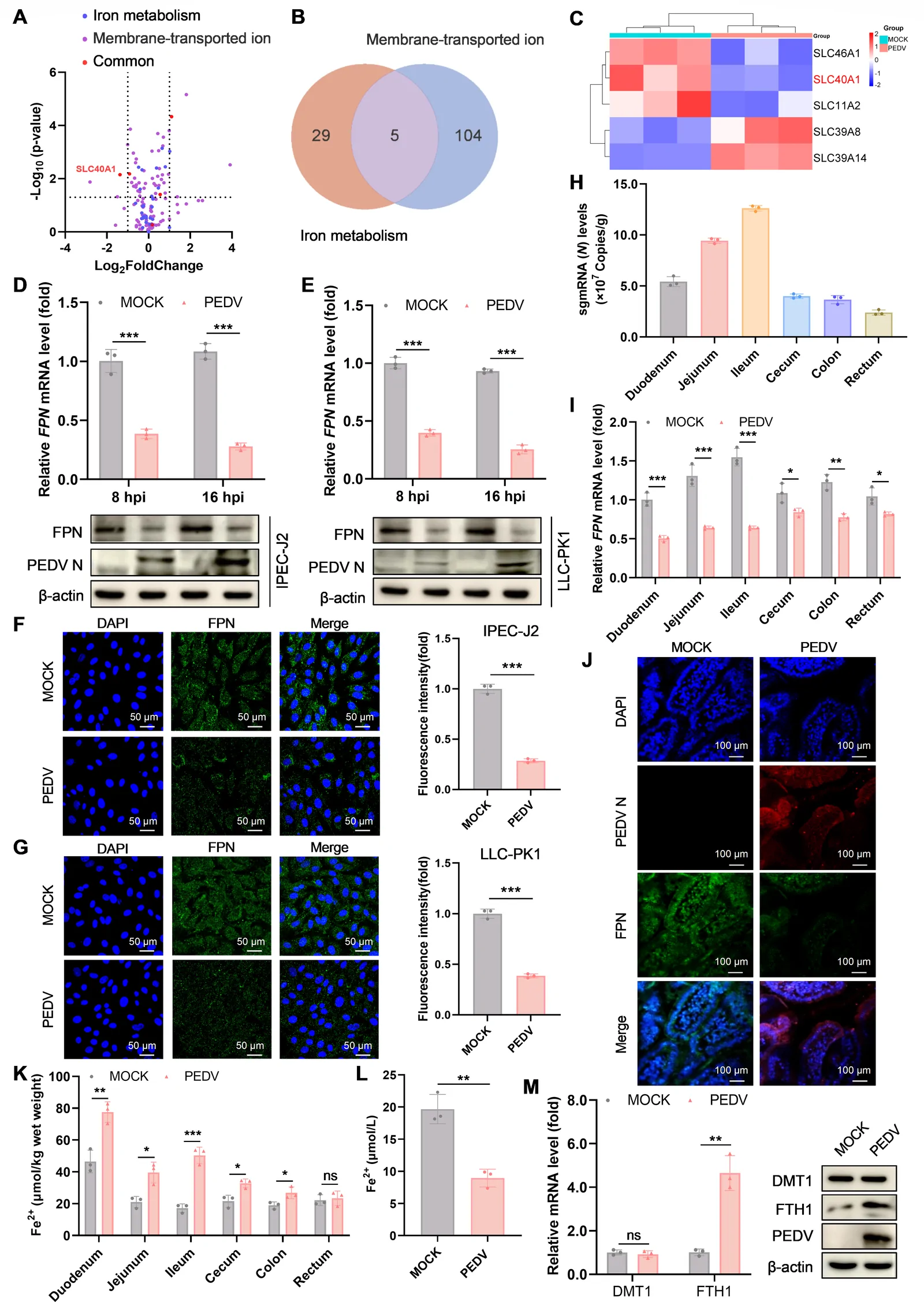
Porcine epidemic diarrhea virus infection suppresses ferroportin expression. (**A–C**) Volcano plot, Venn plot, and heatmap of transcriptome analysis related to iron metabolism and membrane-transported ions in PEDV-infected IPEC-J2 cells (MOI = 1) at 16 h post-infection. (**D and E**) IPEC-J2 and LLC-PK1 cells were harvested for quantitative reverse transcription PCR (qRT-PCR) and western blotting at 8 and 16 hpi following PEDV infection (MOI = 1). qRT-PCR: mean ± SD, *n* = 3 biological replicates with triplicate technical (*n* = 3, triplicate); western blotting: representative, *n* = 3 biological replicates (*n* = 3). (**F and G**) IPEC-J2 and LLC-PK1 cells were infected with PEDV (MOI = 1). At 16 hpi, cells were fixed and incubated with anti-FPN antibody, fluorescently labeled secondary antibody, and DAPI, then visualized by confocal microscopy. Scale bar, 50 µm (*n* = 3). (**H and I**) Piglet intestinal tissues were harvested for qRT-PCR at 7 days post-infection (dpi) following PEDV infection (5 × 10^4^ TCID_50_) (*n* = 3, triplicate). (**J**) At 7 dpi with PEDV (5 × 10^4^ TCID_50_), piglet jejuna were fixed and incubated with anti-FPN, anti-PEDV N antibodies, fluorescently labeled secondary antibodies, and DAPI, then observed by confocal microscopy. Scale bar, 40 µm (*n* = 3). (**K and L**) Piglet intestinal tissues and serum samples were harvested and analyzed by an iron assay kit at 7 dpi following PEDV infection (5 × 10^4^ TCID_50_) (*n* = 3, triplicate). (**M**) IPEC-J2 cells were harvested for qRT-PCR and western blotting at 16 hpi following PEDV infection (MOI = 1) (qRT-PCR: *n* = 3, triplicate; western blotting: *n* = 3). Data were analyzed by a two-tailed Student’s *t*-test (**D–G, I, and K–M**). ns, no significance; *P <  0.05; **P < 0.01; and ***P < 0.001.

To identify potential host antiviral factors against CoVs, we infected neonatal porcine jejunal columnar epithelial IPEC-J2 cells with PEDV (strain LZ202401) at a multiplicity of infection (MOI) of 1 and performed whole-transcriptome sequencing at 16 h post-infection (hpi), focusing on genes associated with membrane ion transport and cellular iron metabolism. Among the differentially expressed genes, *SLC40A1*, encoding a crucial membrane transporter FPN for intracellular iron export to maintain iron homeostasis, was the most significantly downregulated following PEDV infection (Fig. 1A through C). We subsequently verified that the expression of FPN was inhibited by 1 MOI PEDV (strain LZ202401) infection in IPEC-J2 cells and LLC-PK1 cells (Fig. 1D through G).

To assess the expression levels of FPN during PEDV infection *in vivo*, 3-day-old piglets were orally infected with PEDV at a dose of 5 × 10^4^ TCID_50_ and sacrificed at 7 days post-infection. The results showed that FPN was significantly downregulated in the duodenum, jejunum, and ileum, which exhibited the most robust PEDV replication (Fig. 1H through J). Additionally, iron levels were markedly increased in these intestinal segments, whereas serum iron levels were significantly decreased (Fig. 1K and L). These findings suggest that PEDV infection induces altered iron distribution between infected intestinal tissues and the circulation. To determine whether PEDV infection broadly affects iron-related proteins, we further examined other iron transport-related proteins, including ferritin heavy chain (FTH1) and divalent metal transporter 1 (DMT1). Following PEDV infection, FTH1 expression was increased, consistent with an adaptive iron-storage response to elevated intracellular iron levels, whereas DMT1 remained unchanged (Fig. 1M). Together, these data indicate that PEDV infection alters multiple components of the iron-homeostasis network, with FPN showing a prominent reduction among the examined iron-related proteins.

### FPN suppresses PEDV replication by reducing cellular iron availability

**Fig 2:**
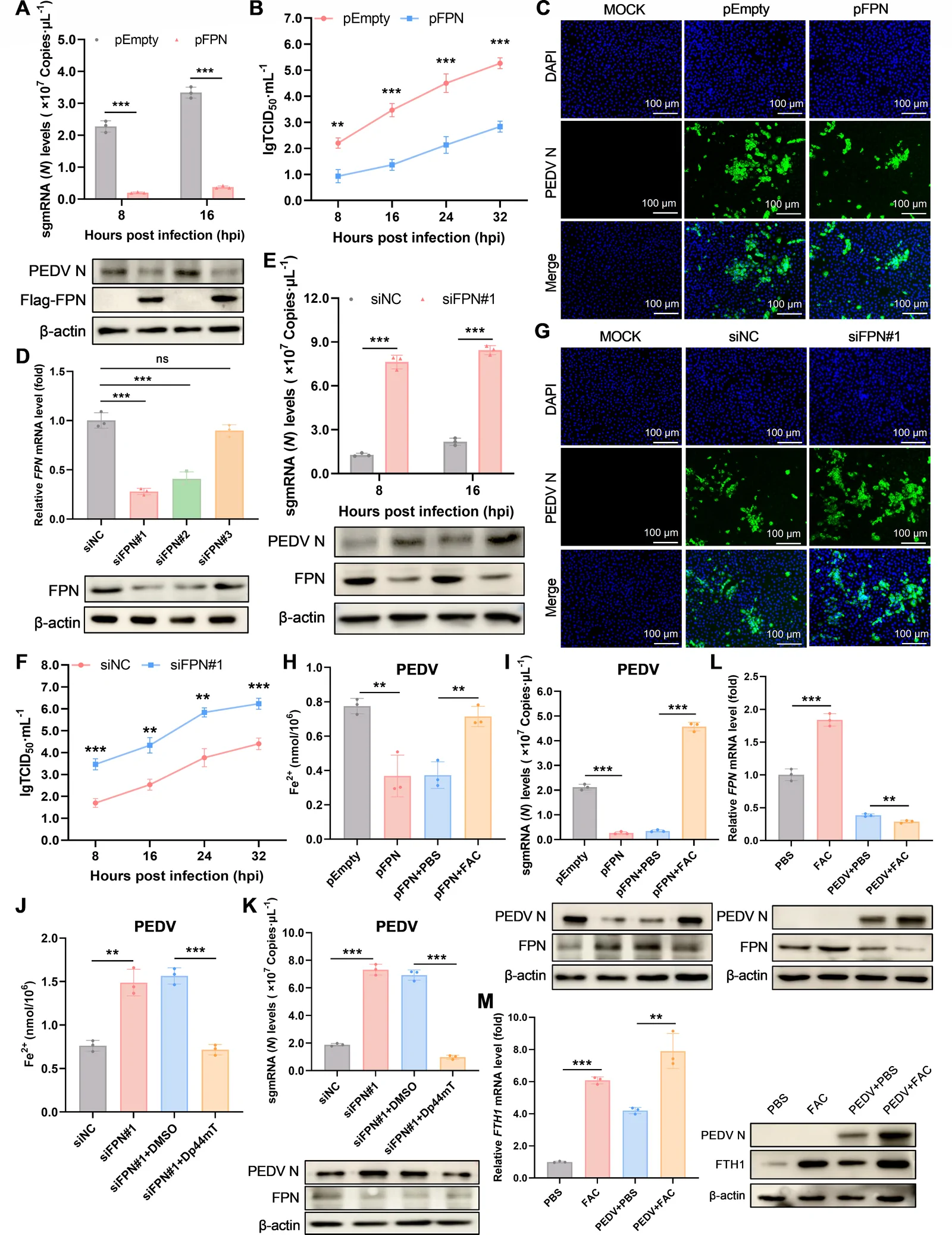
FPN inhibits PEDV replication by downregulating cellular iron. (**A–C**) IPEC-J2 cells were infected with PEDV (MOI = 1) and transfected with Flag-FPN at 12 hpi. At the indicated time post-transfection, cell lysates were collected and analyzed by qRT-PCR, western blotting, TCID_50_, and IFA assay. Scale bar, 100 µm (qRT-PCR, TCID_50_: *n* = 3, triplicate; western blotting, IFA: *n* = 3). (**D**) IPEC-J2 cells were transfected with siFPN, and cell lysates were harvested and analyzed by qRT-PCR and western blotting at 16 h post-transfection (qRT-PCR: *n* = 3, triplicate; western blotting: *n* = 3). (**E–G**) IPEC-J2 cells were infected with PEDV (MOI = 1) and transfected with siFPN#1 at 6 hpi. At the indicated time post-transfection, cell lysates were collected and analyzed by qRT-PCR, western blotting, TCID_50_, and IFA assay. Scale bar, 100 µm (qRT-PCR, TCID_50_: *n* = 3, triplicate; western blotting, IFA: *n* = 3). (**H and I**) IPEC-J2 cells were infected with PEDV (MOI = 1), then transfected with Flag-FPN and treated with ferric ammonium citrate (FAC) at 12 hpi. At 16 h post-treatment, cell lysates were harvested and analyzed by an iron assay kit, qRT-PCR, and western blotting (Fe^2+^, qRT-PCR: *n* = 3, triplicate; western blotting: *n* = 3). (**J and K**) IPEC-J2 cells were infected with PEDV (MOI = 1), then transfected with siFPN#1 and treated with Dp44mT at 6 hpi. At 16 h post-treatment, cell lysates were harvested and analyzed by an iron assay kit, qRT-PCR, and western blotting (Fe^2+^, qRT-PCR: *n* = 3, triplicate; western blotting: *n* = 3). (**L and M**) IPEC-J2 cells were treated with FAC at 12 hpi, either with or without PEDV infection (MOI = 1). At 16 h post-treatment, cell lysates were harvested and analyzed by qRT-PCR and western blotting (qRT-PCR: *n* = 3, triplicate; western blotting: *n* = 3). Data were analyzed by a two-tailed Student’s *t*-test (**A, B, E, F, and H–M**) or one-way ANOVA with Tukey’s multiple comparisons test (**D**). ns, no significance; **P < 0.01; and ***P< 0.001.

To investigate the role of FPN in PEDV infection, IPEC-J2 cells were infected with 1 MOI PEDV and subsequently transfected with Flag-FPN plasmids. Cells and supernatants were collected from the infected cultures. Western blotting, quantitative reverse transcription PCR (qRT-PCR), 50% tissue culture infectious dose (TCID_50_) assay, and immunofluorescence assay (IFA) collectively confirmed that FPN significantly suppresses PEDV replication in IPEC-J2 cells (Fig. 2A through C). To verify these results, FPN-targeting siRNAs were synthesized, and siFPN#1 was selected (Fig. 2D). IPEC-J2 cells were infected with PEDV at an MOI of 1 and subsequently transfected with siFPN#1, and the results showed that decreasing FPN expression significantly facilitated PEDV proliferation (Fig. 2E through G). These findings revealed that FPN plays a crucial role in PEDV infection.

Nevertheless, whether the effect of FPN on PEDV replication depends on its regulation of cellular iron metabolism is still unclear. To verify our hypothesis, we investigated the impact of cellular iron on viral replication by treating IPEC-J2 cells with iron regulators Di-2-pyridylketone-4,4-dimethyl-3-thiosemicarbazone (Dp44mT) and ferric ammonium citrate (FAC) after infection with PEDV at an MOI of 1. Functionally, overexpression of FPN significantly reduced intracellular Fe^2+^ levels and suppressed PEDV replication, an effect that was reversed by FAC treatment (Fig. 2H and I). Conversely, FPN knockdown markedly increased Fe^2+^ levels and promoted PEDV replication, which was abrogated by Dp44mT treatment (Fig. 2J and K). Notably, FAC treatment alone significantly increased FTH1 and upregulated *SLC40A1*/FPN at both the mRNA and protein levels. However, in PEDV-infected cells, the FAC-induced increase in FPN expression was markedly impaired, whereas FTH1 induction was maintained, suggesting that PEDV preferentially disrupts the FPN-mediated iron-export response under iron overload conditions (Fig. 2L and M). Together, these findings suggested that FPN restricts PEDV replication by limiting intracellular iron availability.

### KLF15 directly activates *SLC40A1* transcription and is suppressed by PEDV infection

**Fig 3:**
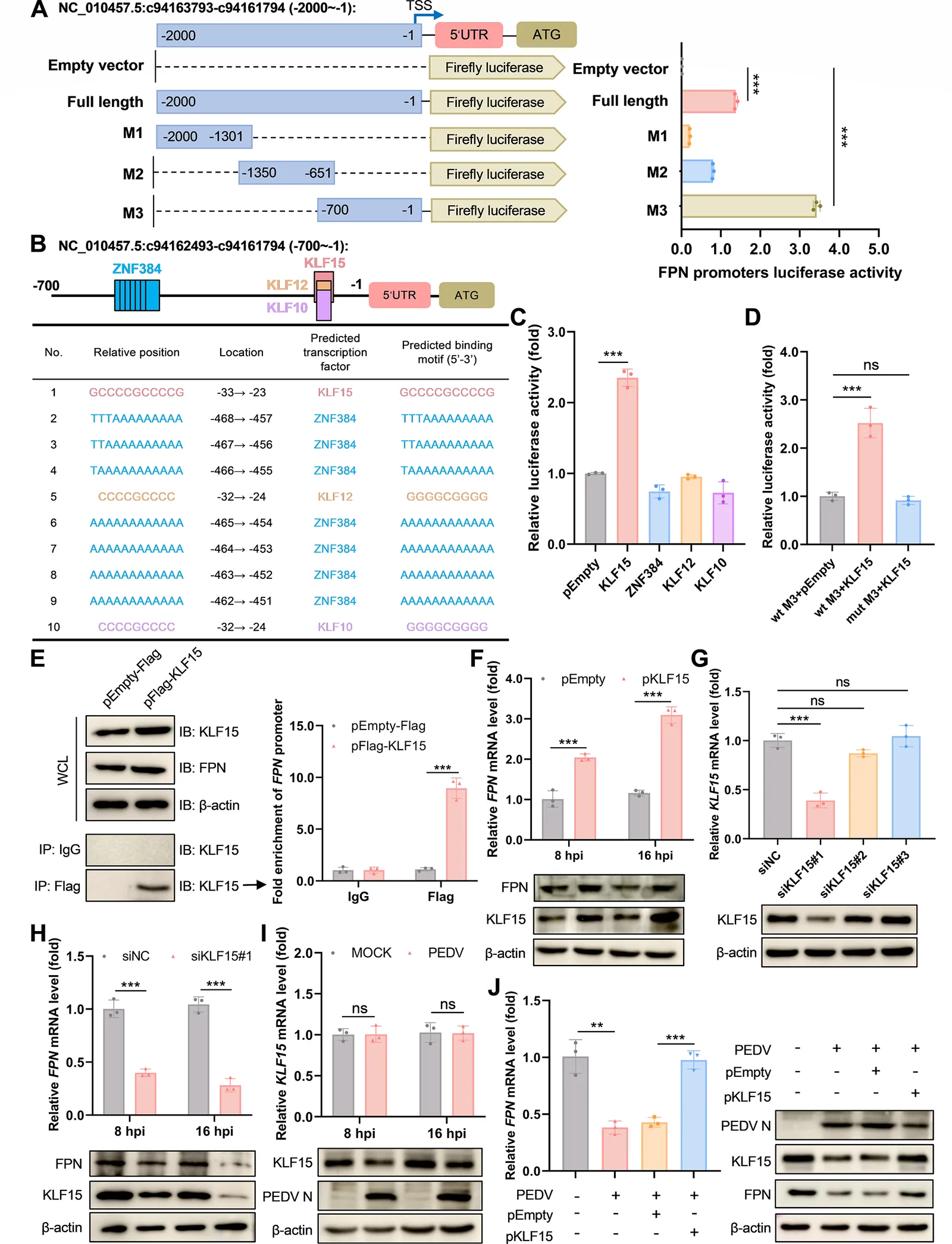
Kruppel-like factor 15 is a key regulatory factor in PEDV-mediated FPN suppression. (**A**) HEK293T cells were transfected with pGL3-Basic vectors containing truncated *FPN* promoter constructs (−2,000 to −1). At 16 hpi, cell lysates were collected, and luciferase activity was assessed (*n* = 3, triplicate). (**B**) Regulatory elements within the −700 to −1 region of the *FPN* promoter predicted by the JASPAR vertebrate database (http://jaspar.genereg.net/). KLF15, ZNF384, KLF12, and KLF10 and their corresponding predicted binding regions are color-coded consistently in the schematic and table. (**C**) HEK293T cells were transfected with the pRL-TK-luc vector, the *FPN* promoter (M3)-driven luciferase vector, and putative transcription factor-encoding plasmids. At 16 hpi, cell lysates were collected, and luciferase activity was assessed (*n* = 3, triplicate). (**D**) IPEC-J2 cells were transfected with the pRL-TK-luc vector, wild-type (wt) or KLF15 binding site mut *FPN* promoter (M3)-driven luciferase vector, and Flag-KLF15 vector. At 16 hpi, cell lysates were collected, and luciferase activity was assessed (*n* = 3, triplicate). (**E**) IPEC-J2 cells were transfected with empty vector or Flag-KLF15. At 16 hpi, cells were analyzed by western blotting (*n* = 3) and chromatin immunoprecipitation, followed by quantitative PCR (ChIP-qPCR) assay (*n* = 3, triplicate). (**F**) IPEC-J2 cells were transfected with Flag-KLF15, and cell lysates were harvested and analyzed by qRT-PCR and western blotting at 16 h post-transfection (qRT-PCR: *n* = 3, triplicate; western blotting: *n* = 3). (**G**) IPEC-J2 cells were transfected with siKLF15, and cell lysates were harvested and analyzed by qRT-PCR and western blotting at 16 h post-transfection (qRT-PCR: *n* = 3, triplicate; western blotting: *n* = 3). (**H**) IPEC-J2 cells were transfected with siKLF15#1, and cell lysates were harvested and analyzed by qRT-PCR and western blotting at 16 h post-transfection (qRT-PCR: *n* = 3, triplicate; western blotting: *n* = 3). (**I**) IPEC-J2 cells were infected with PEDV (MOI = 1) and harvested for qRT-PCR and western blotting at 8 and 16 hpi (qRT-PCR: *n* = 3, triplicate; western blotting: *n* = 3). (**J**) IPEC-J2 cells were infected with PEDV (MOI = 1), then transfected with Flag-KLF15 at 12 hpi. At 16 h post-transfection, cell lysates were harvested and analyzed by qRT-PCR and western blotting (qRT-PCR: *n* = 3, triplicate; western blotting: *n* = 3). Data were analyzed by one-way ANOVA with Tukey’s multiple comparisons test (**A, C, D, and G**) or two-tailed Student’s *t*-test (**E, F, and H–J**). ns, no significance; **P < 0.01; and ***P<0.001.

To identify the cis-regulatory region controlling *SLC40A1* transcription, we generated a series of luciferase reporter constructs containing the full-length *SLC40A1* promoter region and three truncated fragments with 50-bp overlap. The full-length promoter construct covered nucleotides −2,000 to −1 relative to the transcription start site, while the truncated constructs included M1 (−2,000 to −1,301), M2 (−1,350 to −651), and M3 (−700 to −1), respectively (Fig. 3A). These overlapping fragments were designed to systematically cover the promoter region (21, 22). Luciferase reporter assays showed that the M3 fragment exhibited the strongest promoter activity, suggesting that the *SLC40A1* core promoter region lies within −700 to −1 (Fig. 3A). Subsequently, the potential transcription factor binding sites for the *FPN* core promoter were predicted by the JASPAR database (http://jaspar.genereg.net/) (22). Four candidate transcription factors with the highest prediction scores were identified, including Kruppel-like factor 15 (KLF15), Zinc finger protein 384 (ZNF384), KLF12, and KLF10 (Fig. 3B). Among these candidates, KLF15 was validated by luciferase reporter assay to significantly stimulate *FPN* promoter activity (Fig. 3C). Mutation of the KLF15 core consensus sequence C(G/T)CCCC to CAATCC in the *FPN* promoter (M3) (23) significantly decreased its luciferase activity in IPEC-J2 cells (Fig. 3D), indicating that this site is required for KLF15-dependent transcriptional regulation. Chromatin immunoprecipitation followed by quantitative PCR (ChIP-qPCR) confirmed the enrichment of KLF15 at the endogenous *SLC40A1* promoter region (Fig. 3E).

To investigate the regulatory role of KLF15 in FPN expression, IPEC-J2 cells were transfected with Flag-KLF15 or siKLF15. Overexpression of KLF15 significantly upregulated FPN expression, whereas knockdown of KLF15 suppressed it (Fig. 3F through H), indicating that KLF15 functions as a transcriptional activator of *SLC40A1*. Moreover, IPEC-J2 cells were infected with PEDV (MOI = 1) and subsequently transfected with Flag-KLF15. PEDV infection significantly reduced KLF15 protein expression without affecting its mRNA levels (Fig. 3I), whereas overexpression of KLF15 markedly alleviated the PEDV-induced suppression of FPN expression (Fig. 3J), suggesting that PEDV regulates KLF15 at the translational and/or post-translational level.

### PEDV interacts with KLF15 and promotes its cytoplasmic accumulation

**Fig 4:**
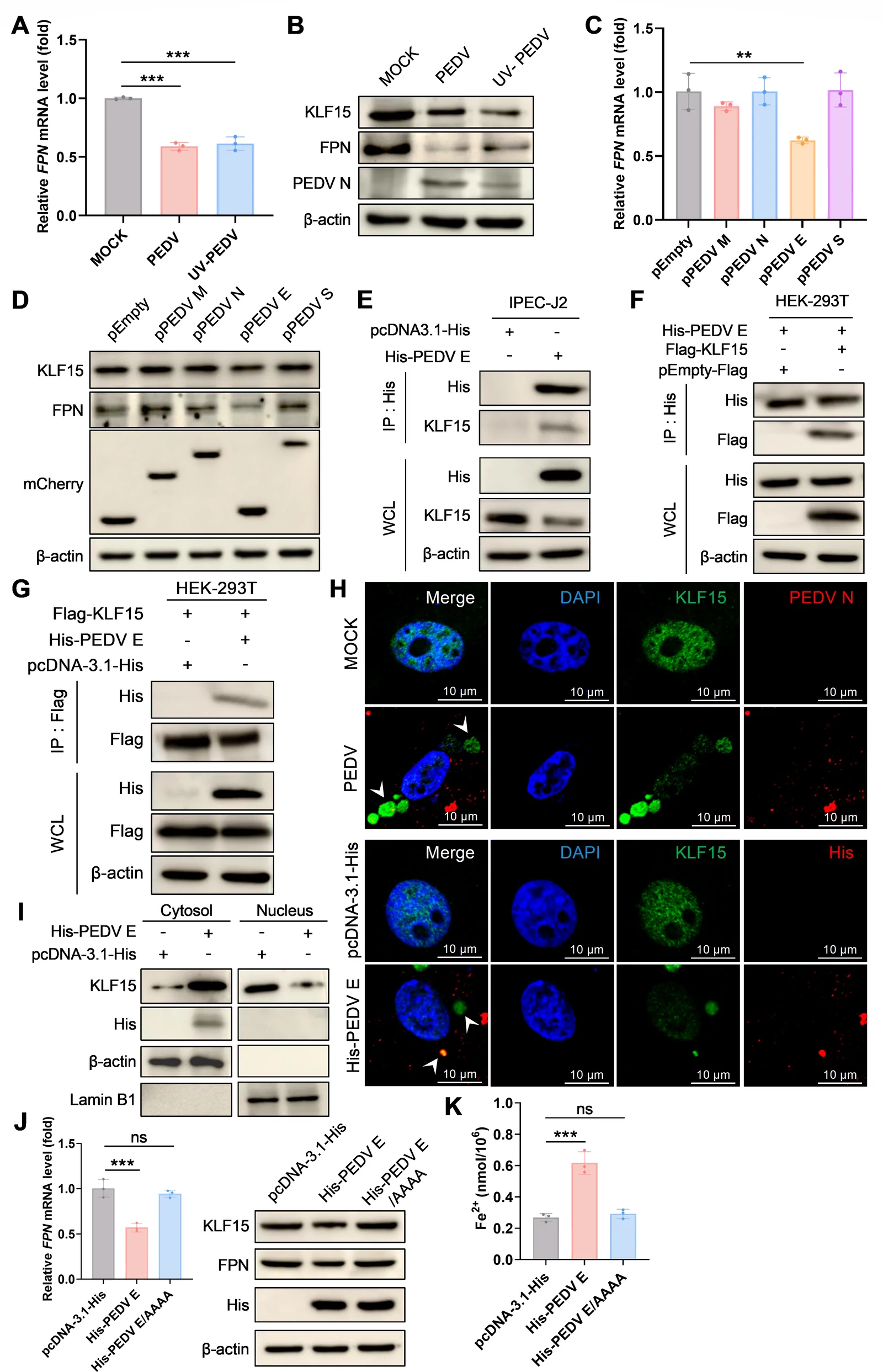
PEDV envelope (E) protein induces KLF15 cytoplasmic aggregation followed by interaction. (**A and B**) IPEC-J2 cells were infected with PEDV or UV-PEDV (MOI = 1) and harvested for qRT-PCR and western blotting at 16 hpi (qRT-PCR: *n* = 3, triplicate; western blotting: *n* = 3). (**C and D**) IPEC-J2 cells were, respectively, transfected with mCherry-tagged PEDV membrane (M), nucleocapsid (N), E, and spike (S) proteins. Cell lysates were harvested and analyzed by qRT-PCR and western blotting at 16 h post-transfection (qRT-PCR: *n* = 3, triplicate; western blotting: *n* = 3). (**E**) His-PEDV E was transfected into IPEC-J2 cells and analyzed for interaction with endogenous KLF15 by immunoprecipitation and western blotting at 16 h post-transfection (*n* = 3). (**F and G**) Flag-KLF15 and His-PEDV E were co-transfected in HEK293T cells, and their interaction was analyzed by Co-IP and western blotting at 16 h post-transfection (*n* = 3). (**H**) IPEC-J2 cells were infected with PEDV (MOI = 1) or transfected with His-PEDV E. At 16 hpi or 16 h post-transfection, cells were fixed and incubated with designated primary antibodies, fluorescently labeled secondary antibodies, and DAPI, then visualized by confocal microscopy. Scale bar, 10 µm (*n* = 3). (**I**) IPEC-J2 cells were transfected with His-PEDV E for 16 h, followed by nucleocytoplasmic fractionation and western blotting analysis. Lamin B1 and β-actin were used as nuclear and cytoplasmic fraction markers, respectively (*n* = 3). (**J and K**) IPEC-J2 cells were transfected with His-PEDV E or the PBM-mutant (His-PEDV E/AAAA, all PBM residues mutated to alanine). At 16 h post-transfection, cell lysates were harvested and analyzed by qRT-PCR, western blotting, and an iron assay kit (qRT-PCR, Fe^2+^: *n* = 3, triplicate; western blotting: *n* = 3). Data were analyzed by one-way ANOVA with Tukey’s multiple comparisons test (**A, C, J, and K**). ns, no significance; **P < 0.01; and ***P < 0.001.

To clarify the mechanism of how PEDV regulates KLF15, we first examined whether productive viral replication is required for KLF15 suppression. Both wild-type and UV-inactivated PEDV (UV-PEDV) reduced FPN mRNA levels and also suppressed KLF15 protein expression (Fig. 4A and B), indicating that suppression of KLF15 and FPN does not require productive viral replication and may involve virion-associated components. We then constructed eukaryotic expression vectors for the M, N, E, and S proteins of PEDV and found that PEDV E protein produced the most pronounced reduction in KLF15 and FPN expression (Fig. 4C and D). Furthermore, co-immunoprecipitation (Co-IP) assays revealed that the PEDV E protein interacts with endogenous KLF15 protein in IPEC-J2 cells (Fig. 4E), while their exogenous interaction was further validated in HEK293T cells (Fig. 4F and G). Given that PEDV E localizes in the cytoplasm while KLF15 resides in the nucleus (24, 25), we next investigated how PEDV E affects KLF15 subcellular localization. The IFA results revealed that PEDV E and KLF15 co-localize in the cytoplasm, indicating that PEDV E induces the cytoplasmic accumulation of KLF15 (Fig. 4H). Nucleocytoplasmic fractionation followed by western blotting showed that expression of PEDV E reduced the nuclear fraction of KLF15 while increasing its cytoplasmic fraction, confirming that PEDV E induces nucleus-to-cytoplasmic redistribution of KLF15 (Fig. 4I). Since the C-terminal PDZ-binding motif (PBM) of CoV E protein is known to interact with host proteins to exert regulatory functions (26, 27), we constructed a mutant plasmid by substituting the endogenous PBM amino acids of the PEDV E protein with alanines (His-PEDV E/AAAA). Compared with wild-type E, the PBM mutant showed markedly reduced ability to suppress KLF15 and FPN expression and increase intracellular Fe^2+^ levels (Fig. 4J and K), demonstrating that the PBM is essential for PEDV E-dependent regulation of the KLF15-FPN-iron pathway.

### PEDV E protein promotes K63-linked ubiquitination and degradation of KLF15

**Fig 5:**
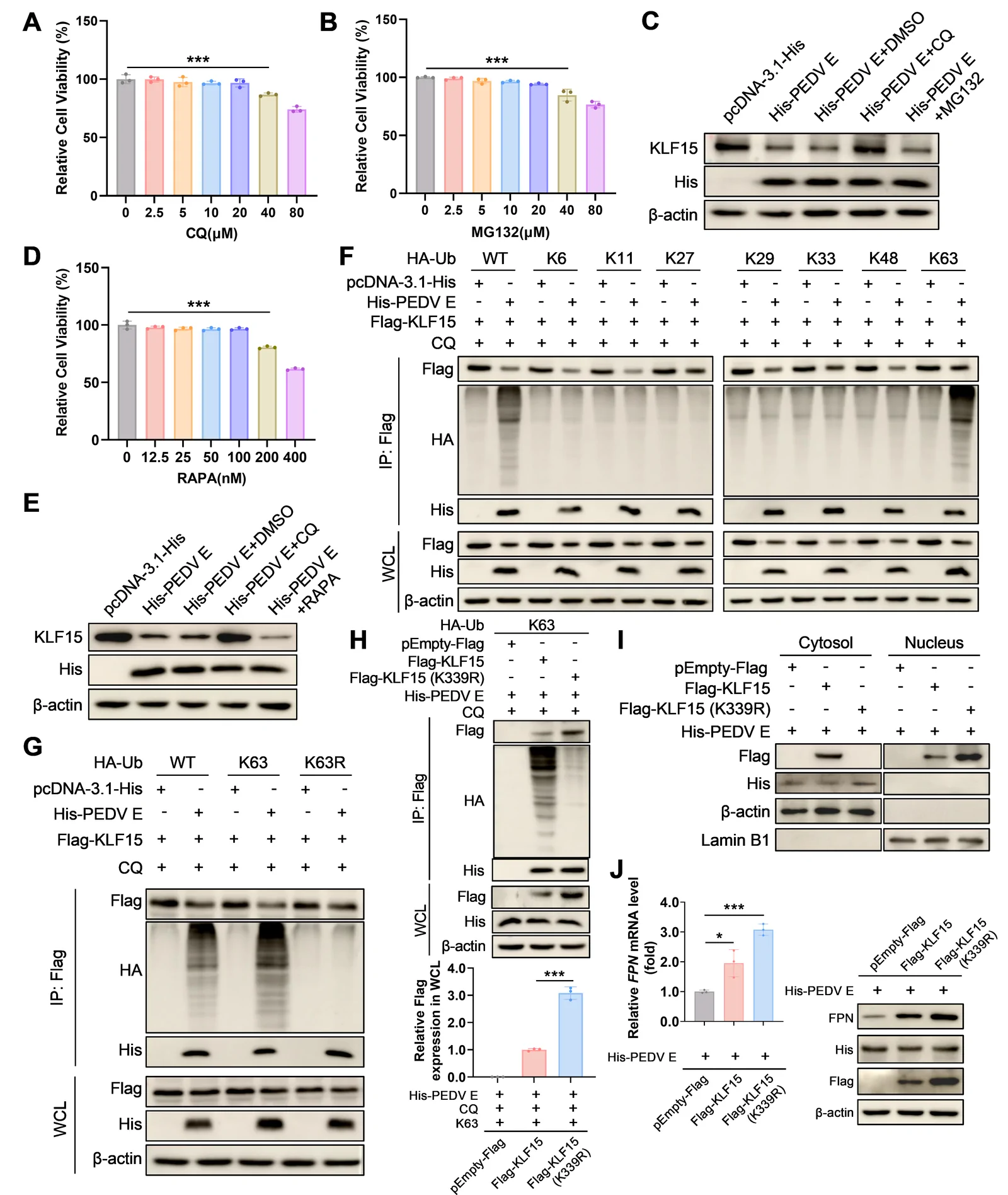
PEDV E protein triggers KLF15 degradation via promoting K63-linked ubiquitination. (**A and B**) IPEC-J2 cells were lysed for cell counting kit-8 assay at 16 h post-chloroquine phosphate and MG132 treatment (*n* = 3, triplicate). (**C**) IPEC-J2 cells were infected with PEDV (MOI = 1), treated with 20 µM CQ or MG132 for 6 h before harvest, and collected at 16 hpi for western blotting analysis (*n* = 3). (**D**) IPEC-J2 cells were lysed for CCK-8 assay at 16 h post-rapamycin treatment (*n* = 3, triplicate). (**E**) IPEC-J2 cells were infected with PEDV (MOI = 1), treated with 100 nM RAPA for 6 h before harvest, and collected at 16 hpi for western blotting analysis (*n* = 3). (**F and G**) HEK293T cells were co-transfected with Flag-KLF15, His-PEDV E, and various ubiquitin subtypes, respectively. Cells were treated with 20 µM CQ for 6 h before harvest and collected at 16 h post-transfection for IP analysis (*n* = 3). (**H**) HEK293T cells were co-transfected with HA-Ub (K63), His-PEDV E, and Flag-KLF15 or Flag-KLF15 (K339R). Cells were treated with 20 µM CQ for 6 h before harvest and collected at 16 h post-transfection for IP analysis (*n* = 3). (**I**) IPEC-J2 cells were co-transfected with His-PEDV E and Flag-KLF15 or Flag-KLF15 (K339R) for 16 h, followed by nucleocytoplasmic separation and western blotting analysis (*n* = 3). (**J**) IPEC-J2 cells were co-transfected with His-PEDV E and Flag-KLF15 or Flag-KLF15 (K339R). At 16 h post-transfection, cell lysates were harvested and analyzed by qRT-PCR and western blotting (qRT-PCR: *n* = 3, triplicate; western blotting: *n* = 3). Data were analyzed by one-way ANOVA with Tukey’s multiple comparisons test (**A, B, D, and J**) or two-tailed Student’s *t*-test (**H**). *P < 0.05; ***P < 0.001.

To determine the pathway responsible for PEDV E-induced KLF15 degradation, cells were treated with the lysosomal inhibitor chloroquine phosphate (CQ) or the proteasome inhibitor MG132. Using the cell counting kit-8 (CCK-8) assay, the non-cytotoxic working concentration of both CQ and MG132 was determined to be 20 μM (Fig. 5A and B). Therefore, 20 μM MG132 and CQ were used for the cell treatment. Our results suggested that the PEDV E-induced KLF15 degradation was inhibited by CQ (Fig. 5C), not by MG132, indicating that PEDV E may degrade KLF15 through the autophagy-lysosome system. To further validate this, we treated cells with the autophagy activator rapamycin (RAPA). A CCK-8 assay identified 100 nM RAPA as a non-cytotoxic concentration under our experimental conditions (Fig. 5D). Treatment with 100 nM RAPA significantly enhanced PEDV E-induced KLF15 degradation, supporting the contribution of autophagy activation to KLF15 degradation (Fig. 5E).

We next investigated the type of ubiquitination modification associated with the degradation of KLF15 mediated by PEDV E. The results showed that PEDV E preferentially mediates the K63-linked polyubiquitination of KLF15 rather than K48-linked ubiquitination (Fig. 5F and G). Furthermore, we searched the PhosphoSitePlus database (https://www.phosphosite.org) (28) and identified lysine 339 (K339) as a potential ubiquitination residue. Mutation of this site from lysine to arginine (K339R) abolished PEDV E protein-induced K63-linked ubiquitination of KLF15 and attenuated subsequent degradation, with the K339R mutant maintaining higher protein abundance than wild-type KLF15 under PEDV E expression conditions (Fig. 5H). Moreover, nucleocytoplasmic fractionation showed that the K339R mutant largely retained nuclear localization compared with wild-type KLF15 upon PEDV E expression, indicating that K339 is required for PEDV E-induced KLF15 nuclear export (Fig. 5I). Importantly, the K339R mutant promoted FPN expression to a larger extent compared to wild-type KLF15 under PEDV E expression conditions (Fig. 5J), suggesting that the K339 site of KLF15 is required for PEDV E-mediated FPN downregulation. Together, these findings support a model in which PEDV E-mediated K63-linked ubiquitination at K339 promotes KLF15 degradation.

### TRIM28 promotes PEDV E-induced KLF15 ubiquitination and degradation

**Fig 6:**
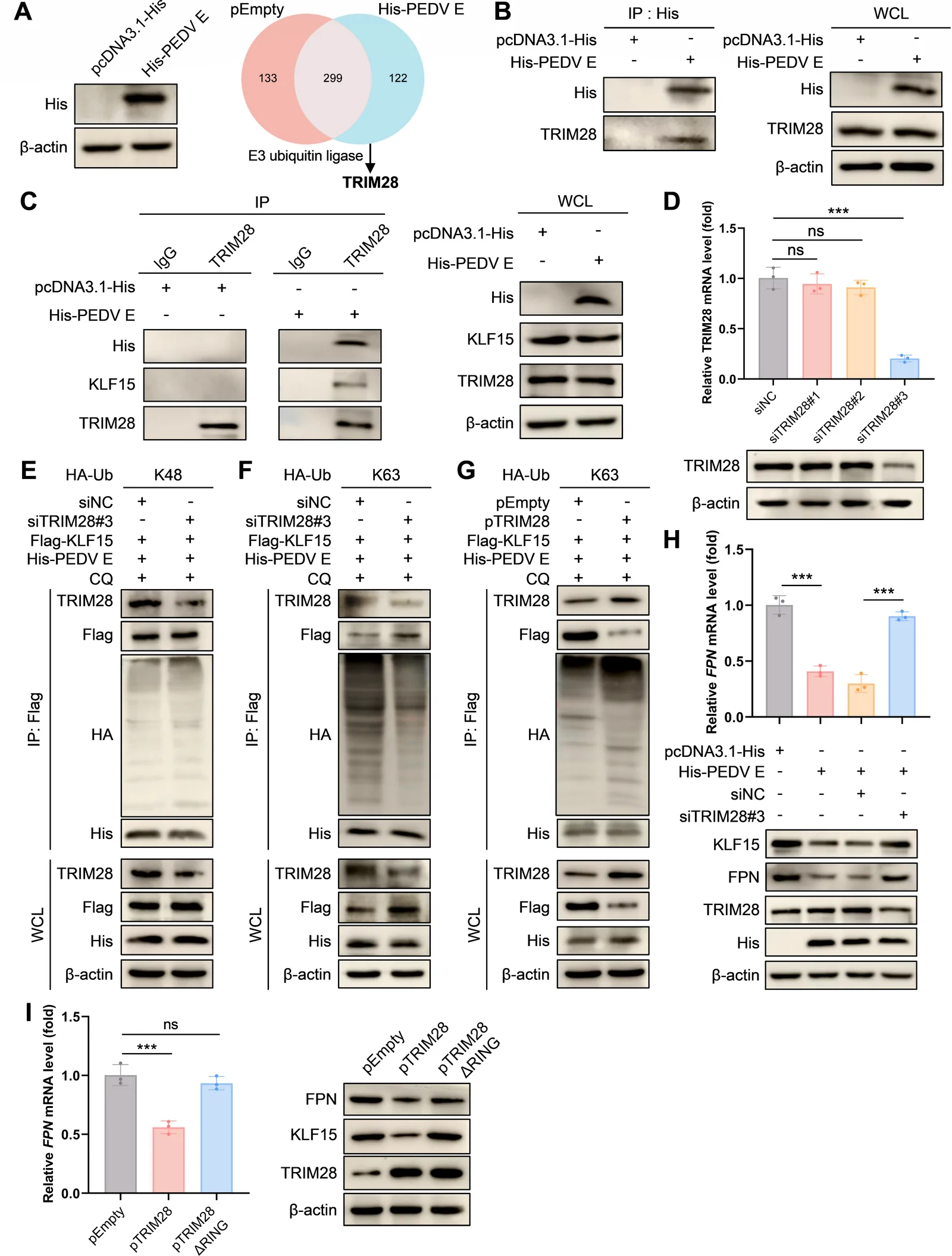
PEDV E protein recruits tripartite motif-containing protein 28 (TRIM28) to degrade KLF15. (**A**) PEDV E-binding proteins identified by IP-MS in IPEC-J2 cells expressing His-PEDV E at 16 h post-transfection (*n* = 3). (**B and C**) His-PEDV E or empty vector was transfected in IPEC-J2 cells, and the interaction with endogenous TRIM28 was analyzed by Co-IP at 16 h post-transfection (*n* = 3). (**D**) IPEC-J2 cells were, respectively, transfected with siNC and siTRIM28. Cell lysates were harvested and analyzed by qRT-PCR and western blotting at 16 h post-transfection (qRT-PCR: *n* = 3, triplicate; western blotting: *n* = 3). (**E–G**) HEK293T cells were co-transfected with HA-Ub (K48) or HA-Ub (K63), His-PEDV E, Flag-KLF15, and siTRIM28#3 or plasmids encoding TRIM28 (pTRIM28). Cells were treated with 20 µM CQ for 6 h before harvest and collected at 16 h post-transfection for IP analysis (*n* = 3). (**H**) IPEC-J2 cells were, respectively, transfected with His-PEDV E, siNC, or siTRIM28#3. Cell lysates were harvested and analyzed by qRT-PCR and western blotting at 16 h post-transfection (qRT-PCR: *n* = 3, triplicate; western blotting: *n* = 3). (**I**) IPEC-J2 cells were transfected with pTRIM28 or the RING deletion mutant (pTRIM28 ∆Ring). At 16 h post-transfection, cell lysates were harvested and analyzed by qRT-PCR and western blotting (qRT-PCR: *n* = 3, triplicate; western blotting: *n* = 3). Data were analyzed by one-way ANOVA with Tukey’s multiple comparisons test (**D and I**) or two-tailed Student’s *t*-test (**H**). ns, no significance; ***P < 0.001.

To identify the E3 ubiquitin ligase involved in PEDV E-induced KLF15 ubiquitination, we performed immunoprecipitation and mass spectrometry (IP-MS) analysis of PEDV E-associated proteins. Among the differentially enriched proteins, TRIM28 was identified as a candidate E3 ubiquitin ligase (Fig. 6A), and its interaction with the PEDV E protein was confirmed by Co-IP assays (Fig. 6B and C). To investigate whether TRIM28 targets KLF15, we first generated TRIM28 knockdown models using three independent siRNAs, and selected siTRIM28#3 with the most efficient reduction of TRIM28 expression at both mRNA and protein levels for subsequent experiments (Fig. 6D).

We next examined whether TRIM28 regulates the ubiquitination pattern of KLF15 by analyzing K48- and K63-linked ubiquitination. TRIM28 knockdown had minimal effects on K48-linked ubiquitination of KLF15 (Fig. 6E) but markedly reduced K63-linked ubiquitination (Fig. 6F). Conversely, TRIM28 overexpression enhanced these effects (Fig. 6G). These results indicate that TRIM28 preferentially promotes K63-linked ubiquitination of KLF15. Consistently, TRIM28 knockdown attenuated the PEDV E-mediated suppression of FPN at both transcriptional and protein levels (Fig. 6H). We further determined whether the E3 ligase activity of TRIM28 is required for KLF15 regulation by generating a TRIM28 mutant lacking the RING domain, which is essential for E3 ubiquitin ligase activity. Deletion of the TRIM28 RING domain abolished its ability to destabilize KLF15 and consequently restored FPN expression (Fig. 6I). These findings support a RING domain-dependent role of TRIM28 in regulating KLF15 stability and the downstream FPN axis.

**Fig 7:**
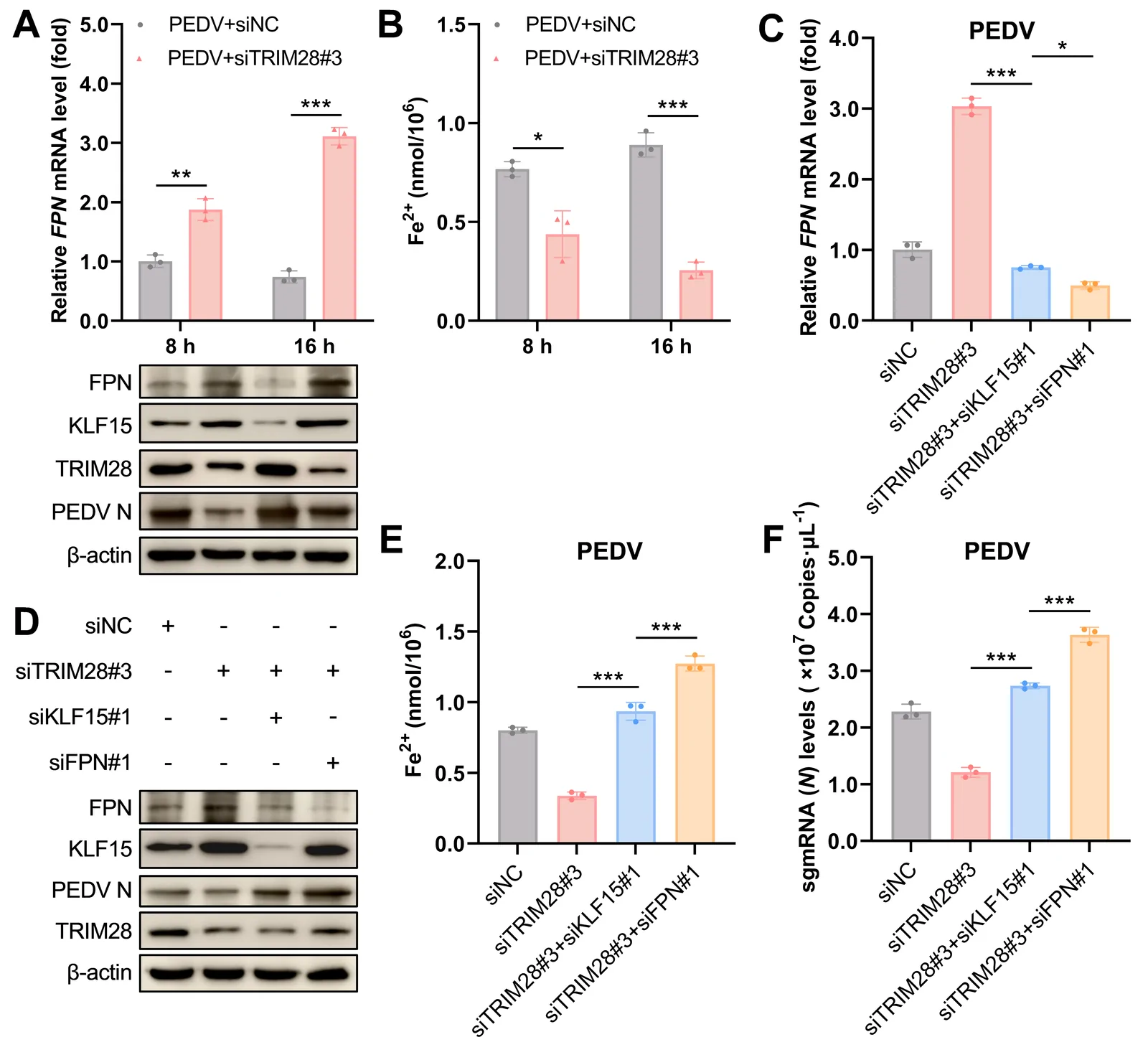
TRIM28 promotes PEDV replication through the KLF15-FPN-mediated iron accumulation pathway. (**A and B**) IPEC-J2 cells were infected with PEDV (MOI = 1), then transfected with siNC or siTRIM28#3 at 6 hpi. At 16 h post-transfection, cell lysates were harvested and analyzed by qRT-PCR, western blotting, and an iron assay kit (qRT-PCR, Fe^2+^: *n* = 3, triplicate; western blotting: *n* = 3). (**C–F**) IPEC-J2 cells were infected with PEDV (MOI = 1), then transfected with siTRIM28#3 or siKLF15#1 at 6 hpi. At 16 h post-transfection, cell lysates were harvested and analyzed by qRT-PCR, western blotting, and an iron assay kit (qRT-PCR, Fe^2+^: *n* = 3, triplicate; western blotting: *n* = 3). Data were analyzed by a two-tailed Student’s *t*-test (**A and B**) or one-way ANOVA with Tukey’s multiple comparisons test (**C, E, and F**). *P < 0.05; **P < 0.01; and ***P < 0.001.

To determine whether the effects of TRIM28 on intracellular iron availability and PEDV replication are mediated through the KLF15-FPN axis, we next performed genetic rescue and epistasis analyses under PEDV infection conditions. TRIM28 knockdown restored KLF15 and FPN expression, reduced intracellular iron, and suppressed PEDV replication (Fig. 7A and B), supporting TRIM28 as an upstream regulator of the KLF15-FPN-iron axis. Moreover, co-depletion of KLF15 abolished the restoration of FPN expression and reversed the reductions in intracellular Fe^2+^ levels and PEDV replication caused by TRIM28 depletion. Similarly, co-depletion of FPN restored intracellular Fe^2+^ levels and PEDV replication following TRIM28 knockdown (Fig. 7C through F). Collectively, these data indicate that the PEDV E protein recruits TRIM28 to promote KLF15 degradation, thereby downregulating FPN expression and enhancing viral replication.

## DISCUSSION

**Fig 8:**
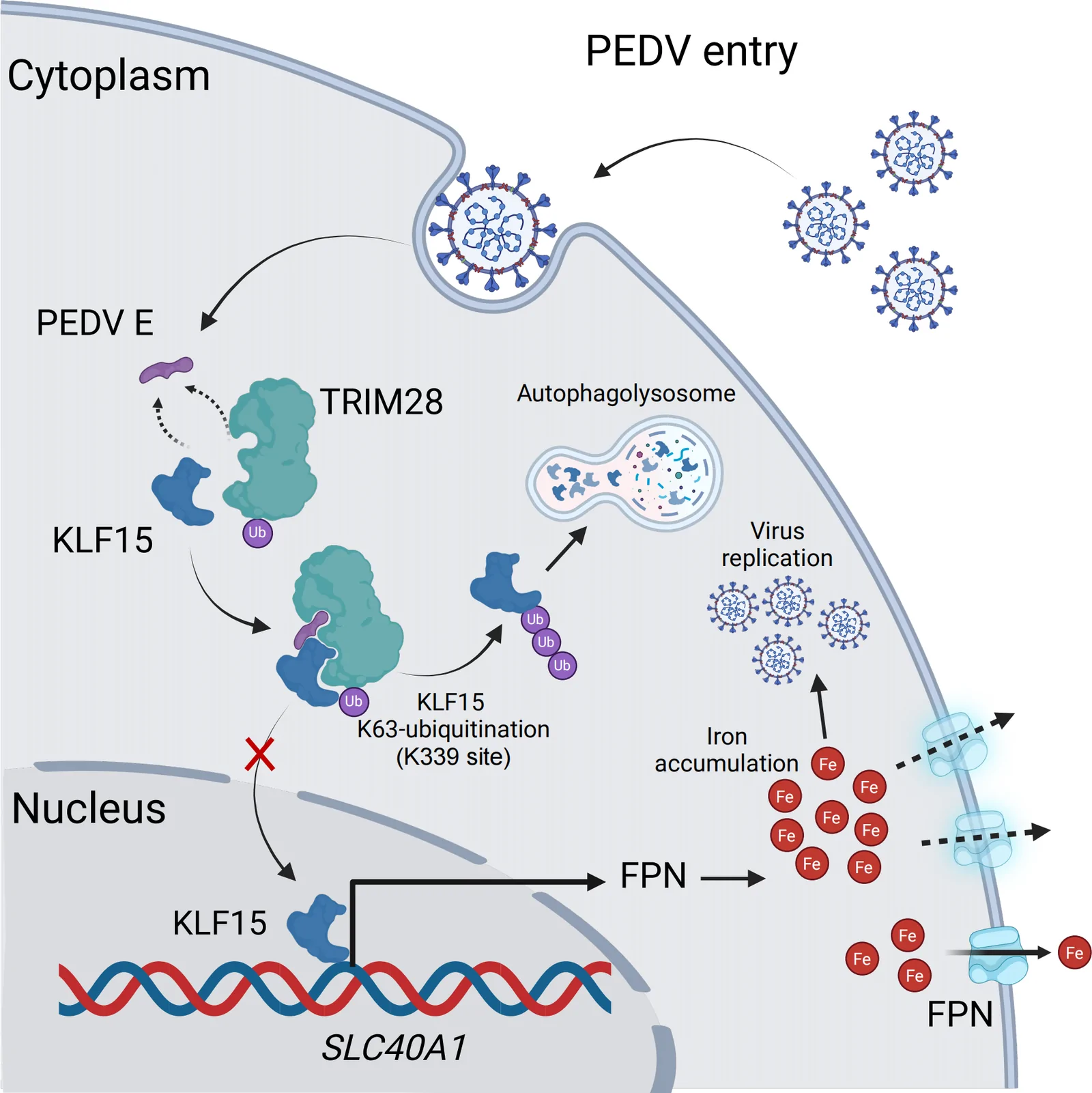
Proposed model of PEDV E-induced intracellular iron retention. Upon PEDV infection, the viral E protein recruits TRIM28, which mediates K63-linked ubiquitination of KLF15 at K339 and its subsequent autophagy-lysosomal degradation. Reduced KLF15 activity suppresses *SLC40A1* transcription and FPN expression, leading to intracellular iron retention and enhanced viral replication.

Iron plays a pivotal role in the viral life cycle, not only participating in fundamental processes such as DNA synthesis and ATP production but also serving as an essential cofactor for many viral proteases, thereby supporting their structural integrity and enzymatic activity (14, 15, 29). Viruses exploit host iron through various strategies, including modulating iron uptake (30–33) or release pathways (34, 35). For instance, SARS-CoV-2 may disrupt iron homeostasis by promoting hemoglobin dissociation and heme release (36). Additionally, HSV-1 and VSV infections degrade the iron exporter FPN via DTX3L upregulation, leading to iron accumulation and immune suppression (20). Despite these advances, whether and how PEDV hijacks host iron remains unclear. Here, we reveal that the PEDV E protein remodels host iron homeostasis by targeting the TRIM28-KLF15-FPN axis, uncovering a previously unrecognized mechanism by which coronavirus alters cellular iron availability to facilitate viral replication (Fig. 8).

Beyond its well-characterized function as an ion channel essential for virion assembly and release (3), our study identifies a novel role for the PEDV E protein in recruiting the E3 ubiquitin ligase TRIM28 to mediate degradation of host factors. Specifically, PEDV E protein facilitates K63-linked ubiquitination and subsequent degradation of the transcription factor KLF15, a newly identified regulator of FPN expression. Mechanistically, PEDV E promotes nuclear-to-cytoplasmic redistribution and destabilization of KLF15, thereby impairing KLF15-dependent transcriptional activation of *SLC40A1*. Our data indicate that cytoplasmic retention of KLF15 is associated with its subsequent degradation; we speculate that PEDV E induces cytoplasmic aggregation of KLF15, which in turn triggers its protein degradation. One possibility of this transport is that E protein may activate cellular signaling pathways, such as the PI3K/Akt or MAPK cascades commonly exploited by CoVs (37–39), to induce KLF15 nuclear export. This hypothesis warrants further investigation. Importantly, *in vivo* analysis in neonatal piglets revealed increased intestinal iron accumulation and reduced serum iron levels following PEDV infection, suggesting infection-associated iron redistribution between infected tissues and circulation. However, whether PEDV induces broader systemic alterations in iron homeostasis remains to be determined.

While our study establishes a mechanistic link between PEDV infection and host iron remodeling, cellular iron metabolism encompasses a complex and finely tuned network. Beyond iron export mediated by FPN, multiple interconnected pathways maintain intracellular iron homeostasis, including iron uptake via transferrin receptor, iron storage within ferritin complexes, and iron utilization in Fe-S cluster biogenesis and heme-containing enzymes (40, 41). Although our functional experiments identify FPN as an important regulator of PEDV-induced intracellular iron accumulation, other iron metabolism regulators, including transferrin receptor 1 and ferritin, were not comprehensively characterized in the present study. Whether PEDV or other CoVs modulate these additional iron metabolic pathways remains to be explored. Given that viruses rely entirely on host resources, future studies integrating metabolomic and proteomic approaches will be necessary to comprehensively map how PEDV reshapes the broader iron homeostasis network.

In summary, our study reveals that PEDV infection disrupts host iron homeostasis through the TRIM28-KLF15-FPN regulatory axis. The PEDV E protein promotes TRIM28-mediated KLF15 ubiquitination and degradation, resulting in reduced *SLC40A1*/FPN expression, intracellular iron retention, and enhanced viral replication. These findings establish a mechanistic link between coronavirus infection and host iron remodeling and highlight the TRIM28-KLF15-FPN axis as a potential target for antiviral intervention. However, the potential risks associated with systemic iron depletion, the specificity, safety, and *in vivo* efficacy of such approaches require further investigation.

## MATERIALS AND METHODS

### Antibodies and reagents

Anti-FPN antibody (26601-1-AP, 1:1,000), anti-FTH1 antibody (11682-1-AP, 1:1,000), anti-DMT1 antibody (20507-1-AP, 1:1,000), anti-KLF15 antibody (66185-1-Ig, 1:2,000), anti-β-actin antibody (81115-1-RR, 1:10,000), anti-mCherry antibody (68088-1-Ig, 1:5,000), anti-Lamin B1 antibody (12987-1-AP, 1:10,000), anti-His-Tag (66005-1-Ig, 1:10,000), anti-Flag-Tag (20543-1-AP, 1:20,000), horseradish peroxidase-conjugated anti-mouse, and anti-rabbit IgG antibodies (SA00001-1 and SA00001-2, 1:10,000) were purchased from Proteintech. Anti-HA-Tag (M20003, 1:5,000) was obtained from Abmart, while anti-TRIM28 antibody (ET1612-55, 1:2,000) was obtained from HUABIO. For immunofluorescence assays, the goat anti-mouse IgG (H + L) Alexa Fluor 488 (SA00013-1, 1:1,000), goat anti-rabbit IgG (H + L) Alexa Fluor 488 (SA00013-2, 1:1,000), and goat anti-mouse IgG (H + L) Alexa Fluor 594 (SA00013-3, 1:1,000) secondary antibodies were sourced from Proteintech. The anti-PEDV N protein antibody was generated in our laboratory as a rabbit polyclonal antibody and used at a 1:500 dilution.

Additionally, Di-2-pyridylketone-4,4-dimethyl-3-thiosemicarbazone (Dp44mT, I339506) was obtained from Aladdin and ferric ammonium citrate (FAC, F5879) from Sigma-Aldrich. MG132 (HY-13259), chloroquine phosphate (CQ, HY-17589A), and rapamycin (RAPA, HY-10219) were from MedChemExpress products.

### Cell culture and virus

Porcine intestinal epithelial (IPEC-J2) cells, African green monkey kidney (Vero) cells, and Lilly Laboratories Culture-Porcine Kidney 1 (LLC-PK1) cells were kindly gifted by Prof. Bin Li (Jiangsu Academy of Agricultural Sciences, China). IPEC-J2 cells were cultured in Dulbecco’s Modified Eagle Medium/Nutrient Mixture F-12 (DMEM/F-12, PM150312, Pricella) supplemented with 10% fetal bovine serum (FBS, 04-001-1ACS, Biological Industries) and 100 µg/mL penicillin/streptomycin. Human Embryonic Kidney 293T (HEK293T) cells (ATCC, CRL-3216), Vero, and LLC-PK1 cells were cultured in DMEM (11965092, Gibco) supplemented with 10% FBS and 100 µg/mL penicillin/streptomycin. All the cell lines were maintained at 37°C and 5% CO_2_ in a humidified atmosphere. The PEDV strain used in this study was the laboratory-isolated LZ202401 (GenBank accession: PP999774). The S gene sequence of PEDV LZ202401 was aligned with representative PEDV strains using DNAMAN software. Phylogenetic analysis was performed using MEGA 7.0 with the maximum likelihood method and 1,000 bootstrap replicates. Based on the phylogenetic analysis, LZ202401 was classified as genotype GIIa.

### Animals

Six 3-day-old SPF piglets (Sus scrofa domesticus) of either sex from a commercial farm in Shaanxi, China, were randomly assigned (by a computer-generated random number table) to mock- or PEDV-infected groups (*n* = 3 per group). All piglets were housed under standard pathogen-free conditions with *ad libitum* access to milk and water. At endpoint, the piglets were euthanized by an overdose of pentobarbital sodium (150 mg/kg of body weight, auricular vein). Intestinal tissues were collected according to predetermined sampling criteria (standardized anatomical locations and uniform weights).

### Plasmids and transfection

The ubiquitin-related vectors used in this study were purchased from Addgene. Partial overexpression vectors and all siRNAs were synthesized by Beijing Tsingke Biotechnology (China), while PEDV structural protein expression vectors were maintained in our laboratory. Other recombinant vectors were constructed by inserting the corresponding exogenous DNA fragments into the indicated vectors via enzymatic ligation or homologous recombination using the one-step cloning kit (RK21020, Genenode). The primers used for plasmid construction are listed in Table 1. For transfection, IPEC-J2, LLC-PK1, or HEK293T cells were cultured to 70% confluency and transfected with recombinant plasmids or siRNAs using the Lipo6000 transfection reagent (C0526, Beyotime).

**TABLE 1:** Sequences of primers and siRNAs used in this study

Purpose	Name	Sequence (5′−3′)
Plasmid construction	Flag-FPN forward	AAGGATGACGATGACAAGCTTATGACCAGGGCAAGAG
	Flag-FPN reverse	TGCCACCCGGGATCCTCTAGATTACACAACAGATGTA
	*SLC40A1* full-length promoter forward	CGCGTGCTAGCCCGGGCTCGAGAAGCCCAGCACTTATAC
	*SLC40A1* full-length promoter reverse	ACAGTACCGGAATGCCAAGCTTTCCTTTCCATTTTGTAAGC
	*SLC40A1* M1 promoter forward	CGCGTGCTAGCCCGGGCTCGAGAAGCCCAGCACTTATAC
	*SLC40A1* M1 promoter reverse	ACAGTACCGGAATGCCAAGCTTACTTTAGCTACTGCTGTT
	*SLC40A1* M2 promoter forward	CGCGTGCTAGCCCGGGCTCGAGGGAAAAAAGAGGGCTTG
	*SLC40A1* M2 promoter reverse	ACAGTACCGGAATGCCAAGCTTTCTTCAGAGGAGAAAATGG
	*SLC40A1* M3 promoter forward	CGCGTGCTAGCCCGGGCTCGAGAGTCACTGCGGGATTTA
	*SLC40A1* M3 promoter reverse	ACAGTACCGGAATGCCAAGCTTTCCTTTCCATTTTGTAAGC
	KLF15 forward	GGATGACGACGATAAGGAATTCATGGTGGACCACTTGC
	KLF15 reverse	AATTAAGATCTGCTAGCTCGAGTCAGGGGCGCGCAGCG
	ZNF384 forward	GGATGACGACGATAAGGAATTCATGGAAGAATCTCACT
	ZNF384 reverse	AATTAAGATCTGCTAGCTCGAGCTACGAGCTGGCCAGG
	KLF12 forward	GGATGACGACGATAAGGAATTCATGAATATCCATATGAAGAGG
	KLF12 reverse	AATTAAGATCTGCTAGCTCGAGTCACACCAGCATGTGC
	KLF10 forward	GGATGACGACGATAAGGAATTCATGCTCAACTTCGGCG
	KLF10 reverse	AATTAAGATCTGCTAGCTCGAGTCACTGCGTGGGAGCA
Real-time PCR primers	PEDV N forward	CTTTGGTGGTAATGTGGCTG
	PEDV N reverse	TCAACAGCTGTGTCCCATTC
	FPN forward	GCTGTGGTTTCATTTCGGGA
	FPN reverse	AGTTCCCTCCAAGGGTTTTGG
	FTH1 forward	GAATTTGCGCTGCACGTGGT
	FTH1 reverse	TGGCTTTCACCTGCTCATGC
	DMT1 forward	CTTCGTCGTGGTTTACGTCC
	DMT1 reverse	TAGAGATGCTTACCGTGTGCC
	KLF15 forward	TCTCCGGTTTCCATGACAGC
	KLF15 reverse	GGCTGGACGACTAGAAGACG
	TRIM28 forward	CCACAAGGACCACCAGTACC
	TRIM28 reverse	TGGCCATCTTGACATCCACC
	GAPDH forward	ACATCATCCCTGCTTCTACTGG
	GAPDH reverse	CTCGGACGCCTGCTTCAC
	ChIP *FPN* promoter forward	CGGGCCCACCGAGACTTTCC
	ChIP *FPN* promoter reverse	TTCGGGTCGTGAATATGCCC
siRNA sequences	siFPN#1 sense	GGAUGGGUCUCCUAUUAUA
	siFPN#1 antisense	UAUAAUAGGAGACCCAUCC
	siFPN#2 sense	GGUGUACAGAACUCAAUGA
	siFPN#2 antisense	UCAUUGAGUUCUGUACACC
	siFPN#3 sense	GAUCAUCACUAUUGCAAAU
	siFPN#3 antisense	AUUUGCAAUAGUGAUGAUC
	siKLF15#1 sense	GCAGCAAGAUGUACACCAA
	siKLF15#1 antisense	UUGGUGUACAUCUUGCUGC
	siKLF15#2 sense	GGAGGAAAUCGAAGAGUUU
	siKLF15#2 antisense	AAACUCUUCGAUUUCCUCC
	siKLF15#3 sense	GCAGCGACCACCUCUCCAA
	siKLF15#3 antisense	UUGGAGAGGUGGUCGCUGC
	siTRIM28#1 sense	GCACUAGCUGUGAGGAUAA
	siTRIM28#1 antisense	UUAUCCUCACAGCUAGUGC
	siTRIM28#2 sense	CGUUGCAGAAGAACACCAA
	siTRIM28#2 antisense	UUGGUGUUCUUCUGCAACG
	siTRIM28#3 sense	CCAAAGACAUUGUGGAGAA
	siTRIM28#3 antisense	UUCUCCACAAUGUCUUUGG

### Reagents and treatment protocols

Di-2-pyridylketone-4,4-dimethyl-3-thiosemicarbazone (Dp44mT, 10 μM in DMSO) and ferric ammonium citrate (FAC, 10 mM in PBS) were added at 2 hpi or 6 h post-transfection and maintained until harvest. CQ (20 μM in DMSO), MG132 (20 μM in DMSO), and RAPA (100 nM in DMSO) were added 6 h before harvest. Control cells received equivalent volumes of PBS or DMSO.

### Quantitative real-time PCR analysis

RNA was isolated from cells using the TriQuick Reagent (R1100, Solarbio). Reverse transcription was performed using Evo M-MLV RT Mix Kit (AG11728, Accurate Biology). Subsequently, qRT-PCR analysis was performed with SYBR Green PCR Master Mix (2×) (4367659, ABI), and the amplification procedure was as follows: initial denaturation at 95°C for 20 s, followed by 40 cycles at 95°C for 15 s and 60°C for 20 s. The primers are listed in Table 1. Relative gene expression was calculated using the 2^−∆∆Ct^ method with GAPDH as the internal reference (42).

### Virus titration

The production of PEDV progeny was measured using the Reed-Muench method (43). Briefly, Vero cells cultured in 96-well plates were inoculated with 10-fold serial dilutions of samples. After 1.5 h of adsorption at 37°C, the inoculum was replaced with fresh DMEM containing 2% FBS. The plates were incubated at 37°C with 5% CO_2_ for 3–5 days, and the cytopathic effect in each well was observed daily. The viral titer was calculated as the 50% tissue culture infectious dose.

### Protein extraction and immunoblotting

Cells were lysed in a mild lysis buffer containing 1% NP-40 (50 mM Tris-HCl [pH 8.0], 150 mM NaCl, and protease inhibitor cocktail) and subsequently denatured in 5× SDS-PAGE loading buffer for 10 min. Equal amounts of protein were electrophoresed by SDS-PAGE and transferred to polyvinyl difluoride (PVDF) membranes (IPVH00010, Millipore). After blocking for 1 h with blocking buffer (5% skim milk powder and 0.1% Tween-20 in PBS), the membranes were incubated with the primary antibodies overnight at 4°C. After washing, the membranes were incubated with HRP-conjugated second antibodies for 1.5 h at room temperature. The conjugated PVDF membranes were detected with enhanced chemiluminescence (PD202, Oriscience).

### Co-immunoprecipitation

Cells were lysed in a mild lysis buffer containing 1% NP-40 (50 mM Tris-HCl [pH 8.0], 150 mM NaCl, and protease inhibitor cocktail). An aliquot of each lysate was retained as the input control. For endogenous immunoprecipitation, normal species-matched IgG was used as the negative control, whereas cells transfected with the corresponding empty vector were used as controls for tag-based Co-IP assays. Antibodies were conjugated with protein A/G magnetic beads (sc-2003, Santa Cruz) in advance and incubated with the cell lysates overnight at 4°C. Immunoprecipitates were obtained using a magnetic rack, washed three times with lysis buffer, eluted in SDS-PAGE loading buffer, and analyzed by immunoblotting.

### Indirect immunofluorescence assay

Cells were fixed with 4% PFA (P6148, Sigma-Aldrich) for 15 min, subsequently permeabilized with 0.1% Triton X-100 (T9284, Sigma-Aldrich) for 10 min, blocked with 5% BSA for 1 h, and incubated with primary antibodies overnight at 4°C. After washing, cells were incubated with secondary antibodies for 1 h at 37°C. Nuclei were stained with DAPI (C1005, Beyotime) for 10 min before mounting. Images were acquired using a confocal microscope (Nikon, Japan) with consistent settings and analyzed using ImageJ for fluorescence quantification.

### Analysis of ferrous ions (Fe^2+^)

The concentration of Fe^2+^ was assessed via the Ferrous Iron Colorimetric Assay Kit (E-BC-K773-M, E-BC-K881-M; Elabscience). Briefly, samples were lysed, and the resulting samples and standard solutions at varying concentrations were, respectively, mixed with the detection reagent. Following incubation at 37°C for 10 min, the absorbance was measured at 593 nm. A standard curve was then generated to calculate the Fe^2+^ concentration in each sample.

### Luciferase reporter assay

HEK293T cells were transiently transfected with the firefly luciferase reporter and the TK-Renilla luciferase internal control plasmid in a 10:1 ratio. After 24 h post-transfection, the cells were lysed and analyzed with a dual-luciferase reporter assay kit (E1910, Promega).

### Ultraviolet inactivation

Active PEDV was added at a multiplicity of infection of 1. For UV inactivation, an aliquot of PEDV corresponding to an input MOI of 1 was calculated from the infectious titer determined before UV treatment and placed in an open 35-mm dish (with a liquid layer ≤2 mm). The dish was exposed to a calibrated 254 nm UVC lamp at an intensity of 0.1 mW/cm^2^ to deliver a target dose of 500 mJ/cm^2^ and gently swirled every 5 min to ensure uniform exposure. Complete inactivation was confirmed by the absence of CPE after three blind passages on Vero cells. Subsequently, active PEDV, UV-PEDV, or maintenance medium alone (mock control) were inoculated into IPEC-J2 cells.

### Chromatin immunoprecipitation assay

According to the manufacturer’s instructions (BK0045-01, ACE), IPEC-J2 cells were cross-linked with 1% formaldehyde and stopped with 0.1 mol/L glycine. Then, the cell lysates were sonicated and subjected to immunoprecipitation using the anti-Flag and anti-rabbit IgG antibodies. After reversal of cross-links, precipitated DNA fragments were recovered and amplified by qPCR with primers listed in Table 1. Meanwhile, the immunoprecipitated samples were heat denatured using 2× SDS loading buffer, and the supernatants were collected for analysis by SDS-PAGE and western blotting.

### Statistical analysis

Prism 9 (GraphPad Software) was used to perform statistical analyses. Data were represented as mean ± SD and analyzed by two-tailed Student’s *t*-test or one-way ANOVA with Tukey’s multiple comparisons test. ns, no significance. *P < 0.05; **P < 0.01; and ***P < 0.001.

## Data Availability

Raw and processed IP-MS data for the identification of PEDV E protein-interacting proteins are available at https://doi.org/10.6084/m9.figshare.32124223. All other data are contained within the article.
